# Supplementary material for: A rice Serine/Threonine receptor-like kinase regulates arbuscular mycorrhizal symbiosis at the peri-arbuscular membrane
Source: Nat Commun. 2018 Nov 8;9:4677. doi: 10.1038/s41467-018-06865-z (PMC6224560; doi:10.1038/s41467-018-06865-z)
Supplement: Supplementary file 1 — Description of Additional Supplementary Files [file 41467_2018_6865_MOESM1_ESM.docx]

**Title:** Dataset 1.
**Description:** Total membrane proteins from maize and rice

**Title:** Dataset 2.
**Description:** Proteins identified from maize and R. irregularis during symbiosis

**Title:** Dataset 3.
**Description:** Proteins identified from rice and R. irregularis during symbiosis

**Title:** Dataset 4.
**Description:** Symbiosis specific proteins identified from maize and rice

**Title:** Dataset 5.
**Description:** Laser capture microdissection transcriptomic analysis

**Title:** Dataset 6.
**Description:** Proteins and transcripts present across all datasets
