# Supplementary material for: A rice Serine/Threonine receptor-like kinase regulates arbuscular mycorrhizal symbiosis at the peri-arbuscular membrane
Source: Nat Commun. 2018 Nov 8;9:4677. doi: 10.1038/s41467-018-06865-z (PMC6224560; doi:10.1038/s41467-018-06865-z)
Supplement: Supplementary file 3 — Supplementary Information [file 41467_2018_6865_MOESM3_ESM.pdf]

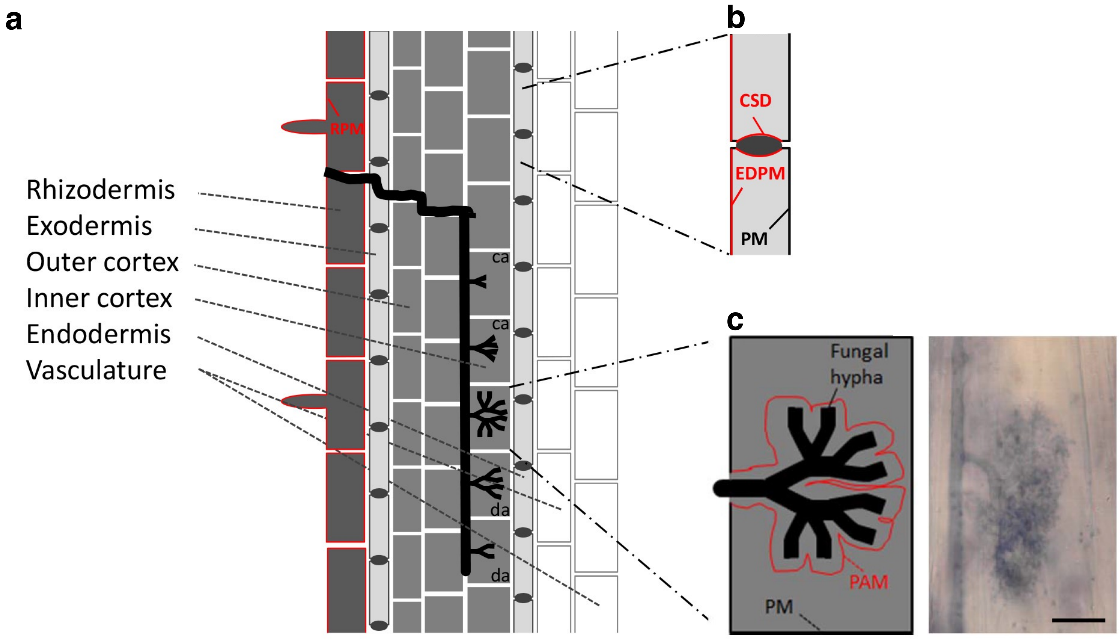

Supplementary Figure 1. Schematic presentation of the cell patterning across the longitudinal axis of the root. Cell types are indicated; ca, collapsing arbuscule; da, developing arbuscule. Membrane subdomains relevant to this study are highlighted in red. **a.** The Rhizodermal Plasma Membrane (RPM). **b.** The Exo-and Endodermal Distal Plasma Membrane (EDPM) domain and the Casparian Strip Domain (CSD) of the exo- and endodermis. **c.** The Peri-Arbuscular Membrane (PAM) envelops the fungal arbuscule during its transient presence within the inner cortex cell. Micrographic presentation of an arbuscule of *Rhizophagus irregularis* within a maize cortex cell. Bar = 10µm.

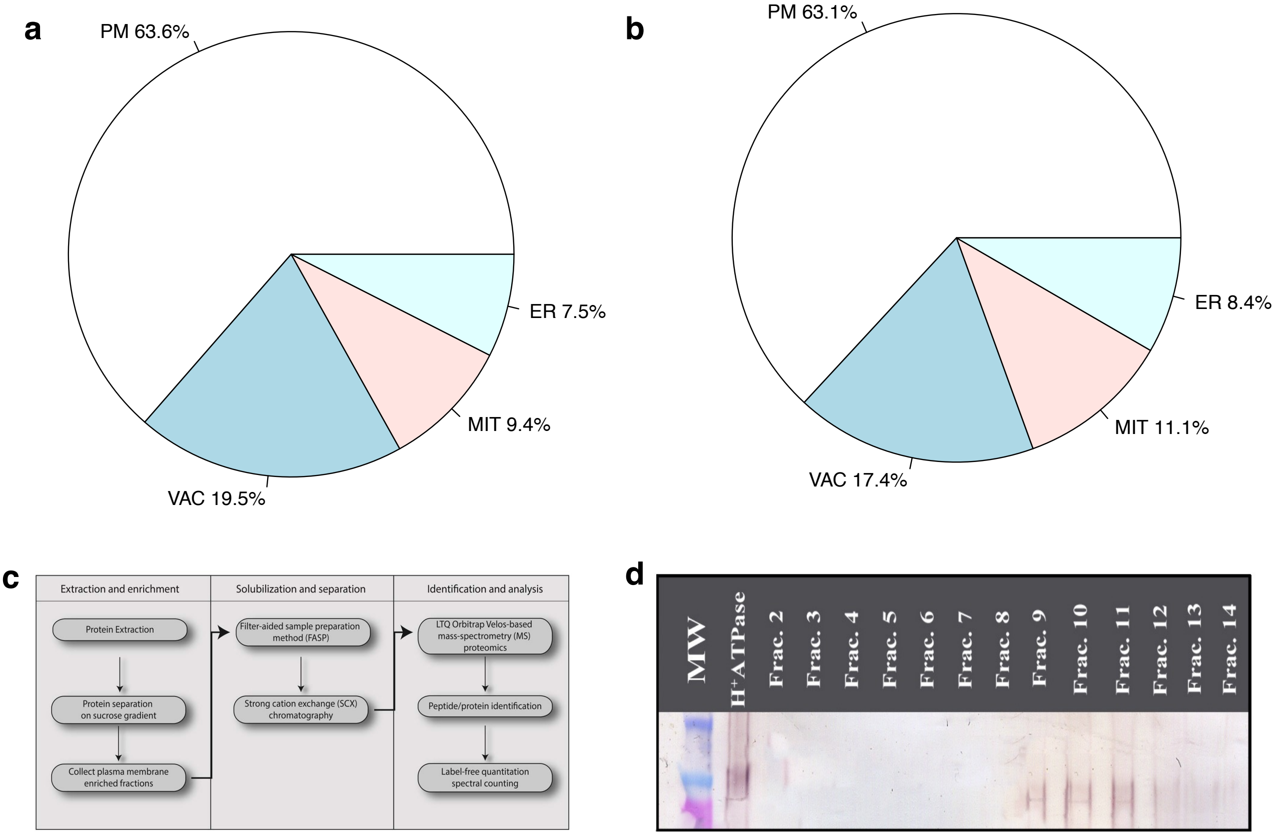

Supplementary Figure 2. Overview over the applied workflow and isolation of membrane proteins a-b. Pie-chart presentation of percentage distribution of membrane proteins of Plasma Membrane (PM, GO:0005886), Vacuole Membrane (VAC, GO:0005774), Endoplasmatic Reticulum Membrane (ER, GO:0005789) and Mitochondria Membranes (MIT, GO:0005743 GO:0005741) for maize and rice, respectively. **c.** Illustration of the sequential steps of the new plasma membrane enriched workflow for proteomics analyses. **d.** Western blot analysis of protein fractions isolated by sucrose gradient. A commercial anti-H^+-ATPase antibody was used. Lane 1, corresponds to the antibody-specific positive control.

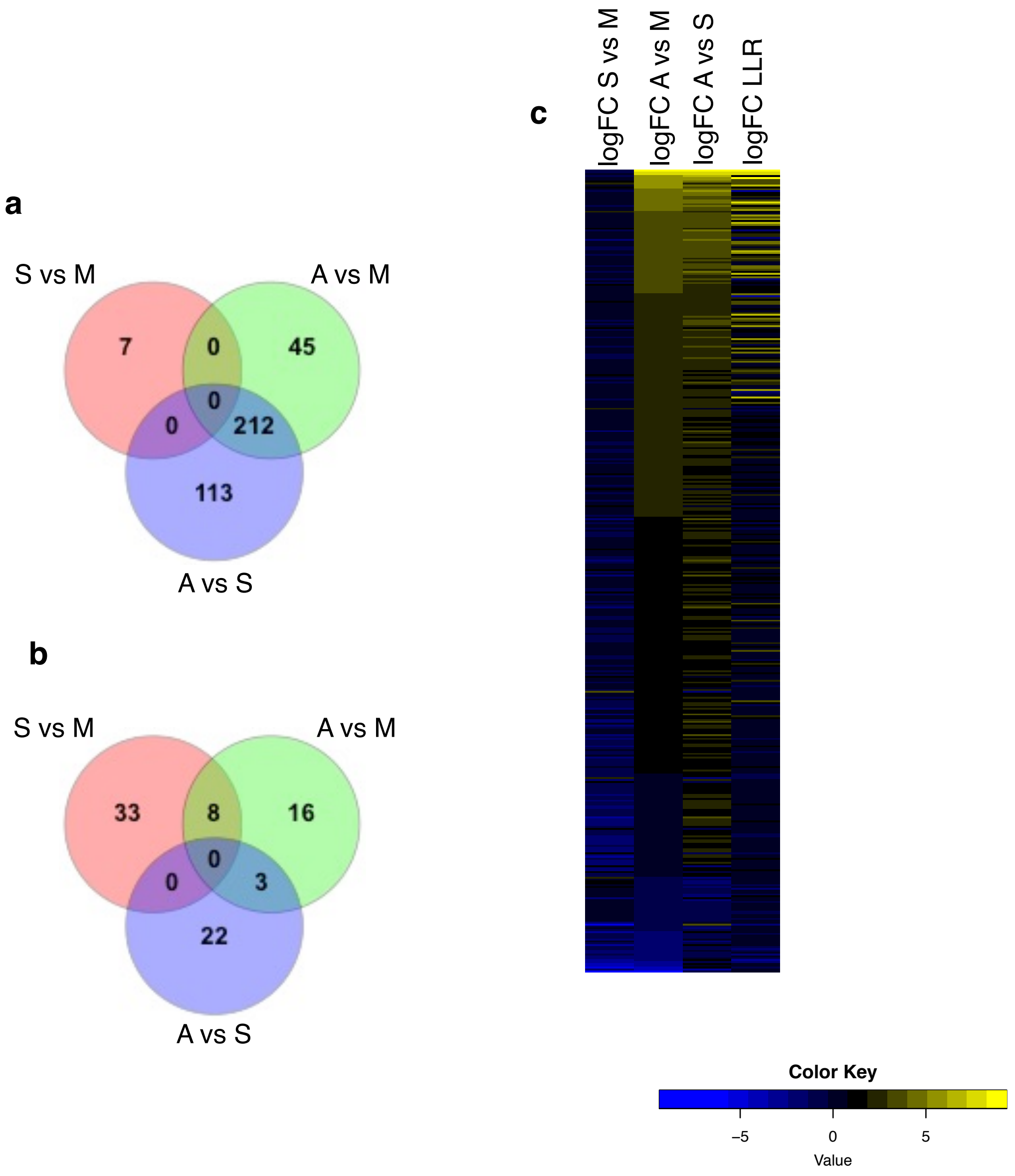

Supplementary Figure 3. Results from the rice cell-specific laser capture micro-dissected (LCM) transcriptome analysis. a. Venn diagram summarising LCM transcriptome data indicating the number of up regulated versus **b.** down regulated transcripts obtained from cortex cells that either contained arbuscules (A), were non-colonized but adjacent to arbusculated cells to monitor systemic induction (S) or were isolated from mock inoculated roots (M). **c.** Heat map showing differentially expressed LCM transcripts obtained from cortical cells containing arbuscules versus cortical cells from mock inoculated roots (A vs M), cortical cells containing arbuscules versus systemically induced transcripts in cortical cells lacking arbuscules (A vs S), systemically induced transcripts in cortical cells lacking arbuscules versus cortical cells from mock inoculated roots (S vs M) for 428 transcripts accumulating differentially in at least one contrast. Also shown are differentially expressed transcripts from colonized versus mock-inoculated large lateral roots (LLR)[1].

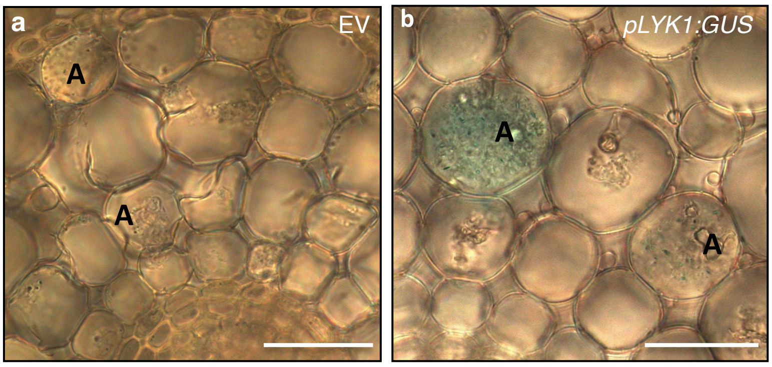

Supplementary Figure 4. *LYK1* promoter activity is specific for arbuscule-containing cells. GUS staining of *R. irregularis* colonized roots at 7 weeks post-inoculation of empty vector (EV) control **(a)** and stable transgenic *pLYK1:GUS* **(b)** lines. **A,** Arbuscule. Scale bar 10µm. Representative images of three roots from five replicate plants are provided.

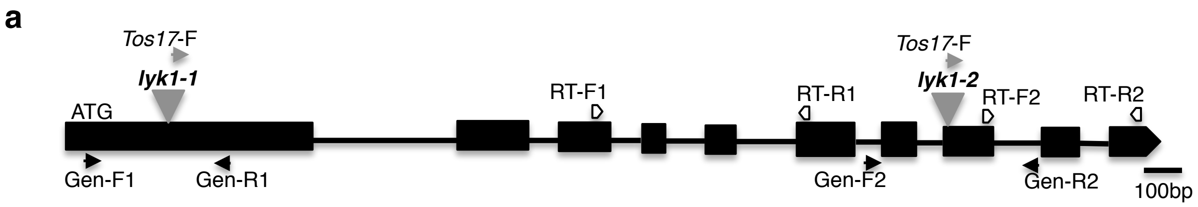

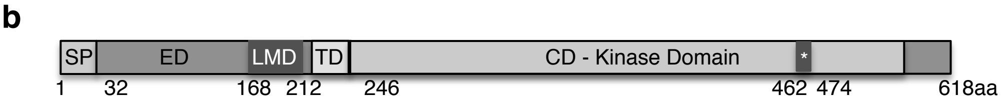

SP MSKHGFLFFLLFFVQHLHTIISLLDSPGGSL

ED SLPEWQPMPCKSVSVNPLCSSYLYVTPEGRNLSKVASDFSG
NASLFQRITRLSGSEDLLVNVPCVCEAINATMTGLFHDTNYR
VKDGDMGDIINSKTFSGLALNVGDGQILHKEEKLIIHLPCGC
SSTAPEGVLS***YAVQDGDTLGNIASLFRSSWKDILDLNPRVAN***
PDFIKPGWILFIPMGVAGPSNKKVRGLP

TM IIIAASISVAMLLLCVLTIILCL

CD KRRSTLPSVEAPAHKMEKIPSNTSIAALESRFYPSMRINEIDPF
QTERPVIFSLRAIEDATSNFDEKRKIGEGGYGSVYLGFIGTHEI
AVKKMKASKSKEFFAELKVLCKIHHINVVELIGYAAGDDHLYL
VYEYVQNGSLSEHLHDPLLKGHQPLSWTARTQIAMDSARG
IEYIHDHTKTCYVHRDIKTSNILLDNGLRAKVADFGLVKL**VQR**
SDEDECLATRLVGTPGYLPPESVLELHMTTKSDVYAFGVVLA
ELITGLRALVRDNKEANKTKSLISIMRKAFKPEDLESSLETIVD
PYLKDNYPIEEVCKLANISMWCLSEDPLHRPEMREVMPILAQ
IHMASIEWEASLGGDGEVFSGVSNGR

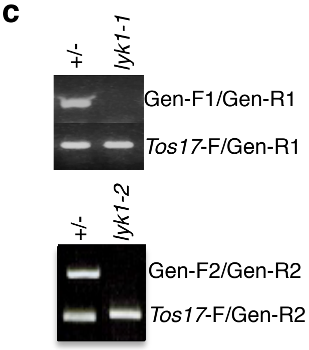

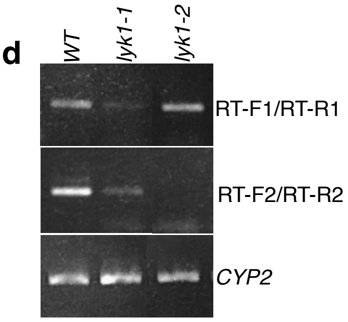

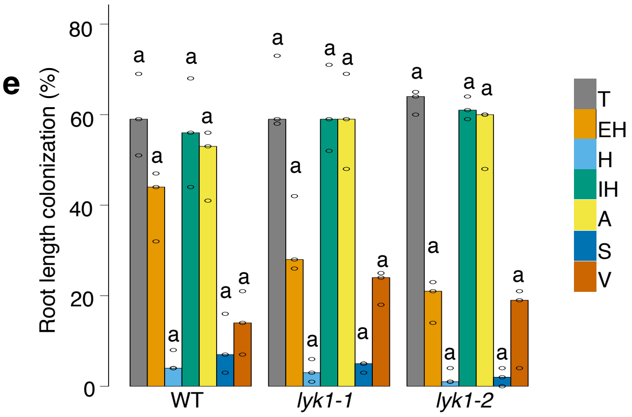

Supplementary Figure 5. *LYK1* gene and protein structure and *R. irregularis* colonization in *lyk1* mutant alleles. a. *LYK1* gene structure with exons boxed and position of *Tos17* transposable element insertions corresponding to each *lyk1* allele; primers used to characterize *lyk1* mutant alleles are indicated. **b**. LYK1 protein structure and sequence with domains indicated by grey boxes; SP, signal peptide; ED, extracellular domain; TM, alpha-helix trans-membrane domain; CD, cytoplasmic kinase domain with activation-loop indicated in bold; repeat Cysteine residue pairs separating Lysine Motif (LysM) domains are underlined. **c.** Confirmation of the presence of *Tos17* retrotransposon in *lyk1-1* and *lyk1-2* mutant allele using Gen-F1/R1 and Gen-F2/R2 to detect the wild-type alleles and *Tos17*-F and Gen-R1/R2 to detect the *Tos17* insertion. +/-, heterozygous plants. *CYP2*, *CYCLOPHILIN2* **d.** Semi-quantitative RT-PCR shows reduced transcript levels in *lyk1-1* and absence of transcripts in *lyk1-2* alleles using primers 3' of the respective *Tos17* insertions (RT-F1 and RT-R1) or 3' of the insertion (RT-F1 and RT-R2). **e**. *R. irregularis* colonization in *lyk1-1* and *lyk1-2* mutant alleles at 6 weeks post inoculation (6wpi). T, Total colonization; EH, extracellular hyphae; H, hyphopodia; IH, intra-radical hyphae; A, arbuscules; S, spores; V, vesicles, open circles correspond to biological replicates. For statistical analysis Kruskal-Wallis tests with the Holm adjustment method were performed using the agricolae with P value set at ≤0.05. Bars show median levels while the letters above each bar indicate colonization values that were not significantly different in the *post hoc* pairwise comparisons.

a

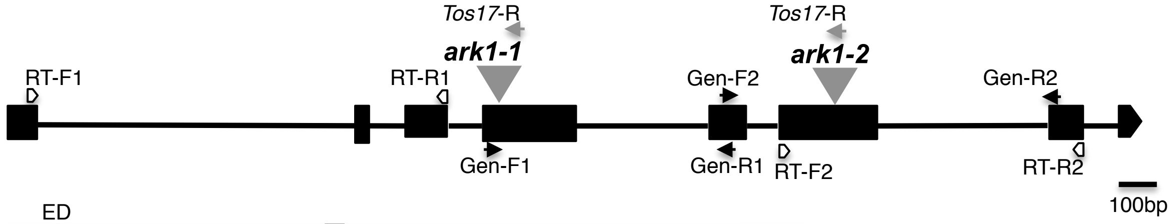

b

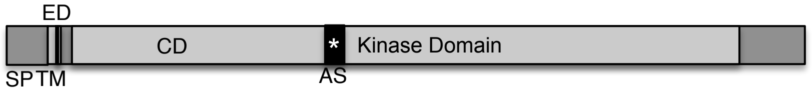

SP MEKVLLWMALIMVTSVGFCKG

ED DQDFVAEGGYIKIKRST

TM FAVVIVFTVMLIALIIALMRYMSKKSKADETIDSTRSSQDNKVHGEVINRWSGLYKFSKGE

CD KKSKADETIDSTRSSQDNKVHGEVINRWSGLYKFSKGEIEKAINYANSKICLGSGSAGQV
YQGVLPSGQLVAIKHIHKSAMSGSFMREVEGLSKVRHPNLVCLFGYCDDGGDQYLVYEY
CANGNLAQNLLRSDSVLSWPARVKILRDCASVLRFLHTHPDGCIVHRDIKLTNILLTESME
PKLSDFGLAKM**LQMEETKVFTDVR**GTIGYMDPEYITHSKLTCASDIYSFGVVALQLLSGR
KVIELDTVARDSLTKKARDVVSGKKPLDEFIDPRVRDEVNIEDFVLILKIAVLCVAHSSVGR
PTIKDVFEEMDKALTNTDSKVGRAREEINPSSTIQYQYATGLNIV

c

Activation loop (AL)

```
i    LjNFR1b 435 VPVYIHRDVKSANILIDKNLRGKVADFGLTKLIEVGNSTL--QTRLVGTFGYMPPE YAQ YGDISPKIDVYAFGVVLFE
     MtLYK3  434 VPVYIHRDVKSANILIDKNLRGKVADFGLTKLIEVGNSTL--HTRLVGTFGYMPPE YAQ YGDVSPKIDVYAFGVVLYE
     LjLYS2  226 VPVYIHRDIKPDNILLNKNFNAKVADFGLTKLTDIESSAINT-DHMAGTFGYMPPE N-A LGRVSRKIDVYAFGVVLYE
     OsCERK1 207 VPVYIHRDIKSANILIDKNYRAKVADFGLTKLTEVGGTSMPTGTRVVGTFGYMPPE YAR YGDVSPKVDVYAFGVVLYE
ii   LjNFR5  424 YPRIIHRDITTSNILLDSNFKAKIANFAMART------------------------ --S TNPMMPKIDVFAFGVLLIE
     AtCerk1 434 VPVYVHRDIKSANILIDQKFRAKVADFGLTKLTEVGGSAT---RGAMGTFGYMAPE T-V YGEVSAKVDVYAFGVVLYE
iii  OsLYK1  360 KTCYVHRDIKTSNILLDNGLRAKVADFGLVKLVQRSDEDECLATRLVGTPGYLPPE SVL ELHMTTKSDVYAFGVVLAE
     OsARK1  119 DGCIVHRDIKLTNILLTESMEPKLSDFGLAKMLQMEETKV--FTDVRGTIGYMDPE YIT HSKLTCASDIYSFGVVALQ
```

Activation segment

d **e**

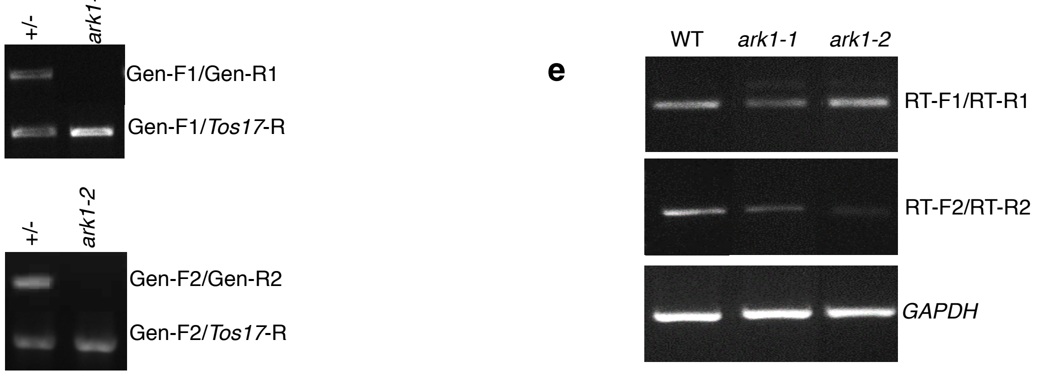

Supplementary Figure 6. ***ARK1*** **gene structure, ARK1 protein sequence and** ***ark1*** **allele analysis** **a.** *ARK1* gene structure with exons boxed and position of *Tos17* transposable element insertions corresponding to each *ark1* allele; primers used to characterize *ark1* mutant alleles are indicated. **b.** ARK1 protein structure and sequence with domains indicated by grey boxes; SP, signal peptide; ED, extracellular domain; TM, alpha-helix trans-membrane domain; AS, kinase activation site; CD, cytoplasmic kinase domain with activation-loop indicated in bold; DFG motif is underlined. **c.** Sequence alignment of ARK1 kinase domains with LYK1, CERK1 homologs as well as the kinase-dead LysM-RLK LjNFR5. Within the activation segment (underlined) the activation loop is highlighted in grey with conserved DFG motif boxed. The symbiosis related CERK1 YAQ/YAR motif is boxed. **d.** Presence of *Tos17* confirmed in *ark1-1* and *ark1-2* mutant alleles using Gen-F1/R1 and Gen-F2/R2 to detect wild-type alleles and Gen-F1/F2 and *Tos17*-R and to detect the *Tos17* insertion. +/-, heterozygous. **e.** Analysis of *ARK1* transcripts accumulation in wild-type (WT) and *ark1* mutant alleles by RT-PCR were carried out in colonized roots at 4 weeks post inoculation (4wpi), using primer pairs located 5' (RT-F1 and RT-R1) and either flanking or 3' of the *Tos17* insertion (RT-F2 and RT-R2). *GAPDH*, *Glyceraldehyde-3-Phosphate Dehydrogenase*.

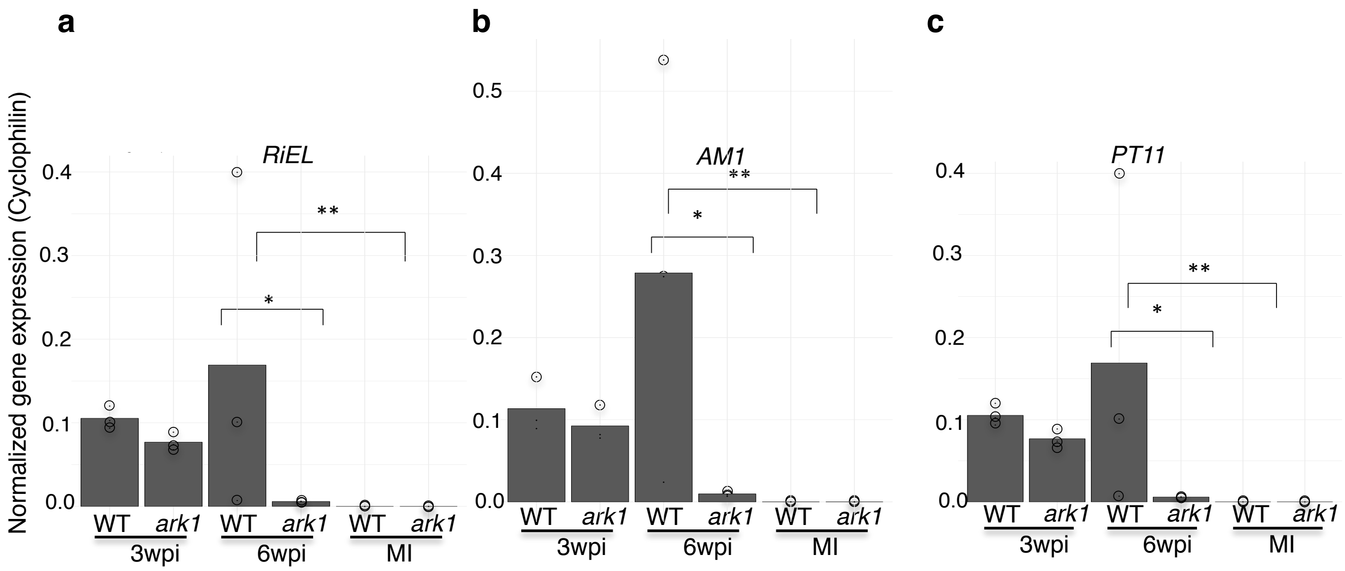

Supplementary Figure 7. AM marker gene expression in the rice *ark1* mutant are reduced at 6 weeks post inoculation (6wpi) compared to wild-type (WT). a. *R. irregularis Elongation Factor* (*RiEL*), **b.** early *AM marker 1* (*AM1*) and **c.** PAM-specific *Phosphate Transporter 11* (*PT11*) marker gene expression at 3wpi and 6wpi. MI, mock inoculated, n=3, * denotes P≤0.05, ** denotes P≤0.005 (Kruskal-Wallis one way ANOVA test). Bars show median levels of gene expression.

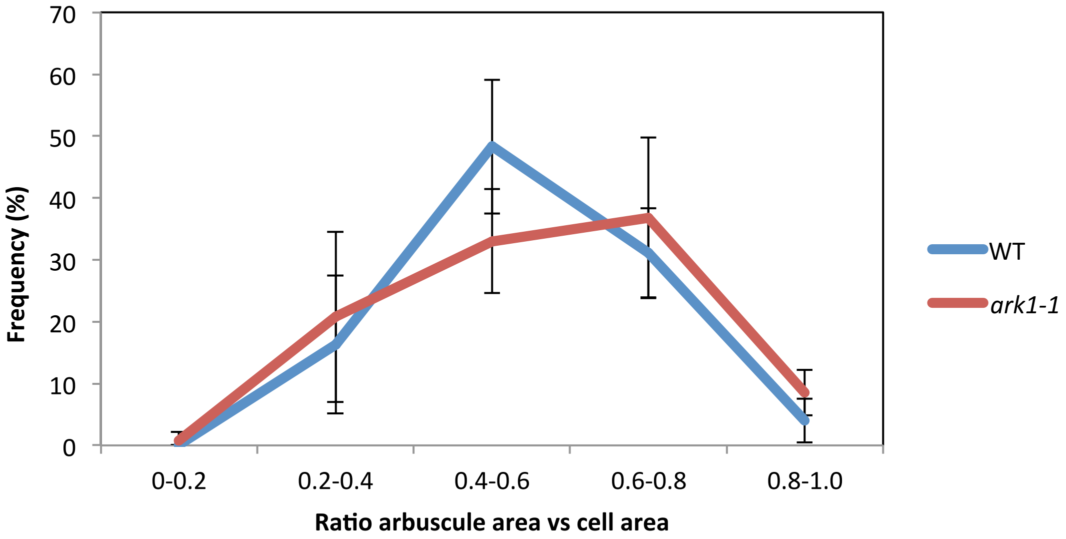

Supplementary Figure 8. Arbuscule size in the *ark1* mutant (n=120) is comparable to that of WT (n=90) at 6 weeks post-inoculation. The circumference of 30 arbuscules were measured per replicate measured as a ratio of the respective cell size. Error bars indicate standard deviation (SD).

Supplementary Table 1. Representative maize marker proteins for plasma membrane, endomembranes and total microsomal fractions as determined by the Scaffold algorithm.

Spectral counts refer to peptides recovered from colonized (Arb) and control (Mock) roots.

			PM enrichment		Total protein fraction		
Identified proteins	ID number	MW	Arb	Mock	Arb	Mock	Reference
Plasma membrane							
H^+- ATPase	GRMZM2G006894	106.0 kDa	406,06	459,24	66,44	104,58	32
PIP1;4	GRMZM2G081843	31 kDa	49,44	50,03	5,09	9,51	33
PIP2;3	GRMZM2G178693	30 kDa	64,17	63,03	16,82	13,89	33
Vacuole							
AZT-type mono-saccharide transporter	GRMZM2G083173	80 kDa	44,18	63,03	10,09	10,70	34
V-ATPase I epsilon subunit	GRMZM2G030167	27 kDa	71,53	78,04	8,49	16,64	35
Endoplasmatic Reticulum							
BiP	GRMZM2G114793	73 kDa	67,33	62,03	34,48	0,00	36
Calreticulin	GRMZM2G305115	55 kDa	5,26	8,00	5,94	2,53	37
Mitochondria							
F0F1	GRMZM5G829375	55 kDa	50,49	48,03	31,96	39,16	38
Tim13	GRMZM2G058432	9 kDa	7,36	5,00	3,36	7,58	39

Supplementary Table 2. List of maize proteins identified at FDR <1% in plasma membrane enriched fractions obtained from mock and colonized root tissue determined by the target-decoy strategy.

Sample	FDR	Total # proteins	Maize proteins	Fungal proteins	Quantifiable proteins	Quantitative correlation
ZmMPM	<1%	3640	3622	18	2953	0.94
ZmAPM	<1%	4192	3568	624	3300	0.90

MPM, Mock plasma membrane proteome; APM, Arbuscular plasma membrane proteome; FDR: False Discovery Rate; Total # proteins: total number of proteins; Quantitative correlation: Pearson correlation between replicates quantitative values

Supplementary Table 3. Representative rice marker proteins for plasma membrane, endomembranes and total microsomal fractions as determined by the Scaffold algorithm.

Spectral counts refer to peptides recovered from colonized (Arb) and control (Mock) roots.

			PM enrichment		
Identified proteins	ID number	MW	Arb	Mock	Reference
Plasma membrane					
H^+- ATPase	LOC_Os04g56160	105 kDa	104.10	116.56	32
PIP1;4	LOC_Os02g57720	31 kDa	14,53	10,74	33
PIP2;3	LOC_Os04g44060	30 kDa	13,32	93,94	33
Vacuole					
AZT-type mono-saccharide transporter	LOC_Os10g39440	79 kDa	12,29	11,1	34
V-ATPase I epsilon subunit	LOC_Os01g46980	27 kDa	42,15	30,87	35
Endoplasmatic Reticulum					
BiP	LOC_Os02g02410	73 kDa	32,68	55,51	36
Calreticulin	LOC_Os05g43170	50 kDa	35,13	0	37
Mitochondria					
F0F1	LOC_Os09g08910	55 kDa	29,27	18,79	38
Tim13	LOC_Os02g45820	9 kDa	64,40	55,51	39

**Supplementary Table 4. Identification of cell-type specific marker proteins in mock control and arbuscule-containing rice roots.**							
Accession number	Description	Membrane subdomain	Highest number of unique peptides MPM	Quantitative value MPM	Highest number of unique peptides APM	Quantitative value APM	Reference
LOC_Os02g43410	Iron-phytosiderophore transporter yellow stripe 1 (YS1)	RPM	8	12,08	6	72,63	26
LOC_Os04g58750	Uncharacterised protein (CASP1-like protein)	CSD	2	4,16	3	36,31	27
LOC_Os01g46860	Inorganic phosphate transporter 11 (PT11)	PAM	0	0	10	18,15	29
LOC_Os09g23640	ABC-2 type transporter domain containing protein (STR1)	PAM	0	0	7	96,84	11

RPM: Rhizodermal plasma membrane; EDPM: Exo- and endodermal; CSD: Casparian strip membrane domain; PAM: Peri-arbuscular membrane; MPM: Mock Plasma Membrane proteome; APM: Arbuscular Plasma Membrane proteome

Supplementary Table 5. List of rice proteins identified at FDR <1% in plasma membrane enriched fractions obtained from mock and colonised root tissue determined by the target-decoy strategy.

Sample	FDR	Total # proteins	Rice proteins	Fungal proteins	Quantifiable proteins	Quantitative correlation
OsMPM	<1%	2036	2008	28	1834	0.93
OsAPM	<1%	3550	2959	591	2383	0.75

MPM, Mock plasma membrane proteome; APM, Arbuscular plasma membrane proteome; FDR: False Discovery Rate; Total # proteins: total number of proteins; Quantitative correlation: Pearson correlation between replicates quantitative values

**Supplementary Table 6. Primers used in this study**		
**Genotyping Primers**		
WT	Fwd	GAGGTGATCAACAGGTGGTCAGG
	Rev	GATAACTTGGGCTCCATGCTCTCT
ark1-1	TOS17F	GACAACACCGGAGCTATACAAATCG
WT	Fwd	GGGATTGTGCATCTGTCTTGAGG
	Rev	CCGCAATTTTCAATATCAACACGA
ark1-2	TOS17F	GACAACACCGGAGCTATACAAATCG
WT	*Lyk1*-F1	GTCCAAGCATGGTTTCCTGT
	Lyk1-R1	TTGCCCAAGGTATCTCCATC
lyk1-1	TOS17F	ATTGTTAGGTTGCAAGTTAGTTAAGA
WT	*Lyk1*-F2	TTACCAGGAGGGAGGTGATG
	Lyk1-R2	AACCAAACGAGTTGCCAGAC
lyk1-2	TOS17F	ATTGTTAGGTTGCAAGTTAGTTAAGA
hptII	HygF-UP	GTTTATCGGCACTTTGCATCGGCCG
	HygR-UP	GATTTGTGTACGCCCGACAGTCC
**RT-PCR Primers**		
LYK1 allele characterisation	RT-F1	TGGTGAGGGAGGATATGGAAG
	RT-R1	CAATACCACGTGCAGAGTCC
	RT-F2	TTACTGGTCTCCGTGCACTT
	RT-R2	GAAGACTTCACCATCGCCTC
ARK1 allele characterisation	RT-F1	GGATGGCTTTGATTATGGTAAC
	RT-R1	GCGTAGTTTATCGCCTTCT
	RT-F2	CTTACTGACAGAGAGCATGGAG
	RT-R2	AGCCCCGTTGCATACTGATACT
AM1	*AM1-F*	TTTGCTTGCCACACGTTTTAA
	AM1-R	ACCTCGCCAAAATATATGTATGCTATT
PT11	*PT11-F*	CATATCCCAGATGAGCGTATCATG
	PT11-R	GAGAAGTTCCCTGCTTCAAGCA
RiEL	*RiEL-F*	GCACCAGTGCTCGATTGC
	RiEL-R	TCGCCTGTCAATCTTGGTAACAA
GAPDH	GAPDH-F	AGGTTCTTCCTGATTTGAATGG
	GAPDH-R	CAACTGCACTGGACGGCTTA
CYCLOPHILIN2	Cyp2-F	TCCCAGTTCTTCATCTGCAC
	Cyp2-R	GCGATATCATAGAAGCAGCGAC
UBIQUITIN	Ubi-F	CATGGAGCTGCTGCTGTTCTAG
	Ubi-R	CAGACAACCATAGCTCCATTGG
**Cloning Primers**		
LYK1	*LYK1* Prom-F	CTCCAAAGTCCCTTGGATGAC
	LYK1 Prom-R	GGTGAGCCACAGGGAAGGCAA
	LYK1-R	TCTACCATTGGAAACGCCACTGAA
ARK1	*ARK1* Prom-F	CTTTGATGGTGTAATGACTAAC
	ARK1 Prom-R	TTCAGCTTCTTCAGTGTCCTCGA
	ARK1-R	TACTATGTTAAGCCCCGTTGC

Supplementary References

1 Gutjahr, C. *et al.* Transcriptome diversity among rice root types during asymbiosis and interaction with arbuscular mycorrhizal fungi. *Proceedings of the National Academy of Sciences of the United States of America* **112**, 6754-6759, doi:10.1073/pnas.1504142112 (2015).
